# Time/Molecular Weight Superposition to Describe the Behavior of Controlled-Rheology Polypropylenes

**DOI:** 10.3390/polym14163398

**Published:** 2022-08-19

**Authors:** Françoise Berzin, Bruno Vergnes

**Affiliations:** 1 Fractionnement des Agro-Rsources et Environnement, University of Reims Champagne Ardenne, INRAE, 51100 Reims, France; 2 CEMEF, UMR CNRS 7635, Mines Paris—PSL, CS 10207, 06904 Sophia-Antipolis, France

**Keywords:** polypropylene, peroxide, molecular weight, viscosity, mastercurve

## Abstract

Polypropylene resins issuing from polymerization reactors are degraded by peroxides in subsequent reactive extrusion processes to improve their processability. This operation reduces their molecular weight and, thus, their viscosity and elasticity. In a previous study, a series of homo- and copolymer polypropylenes of different molecular weight distributions were prepared by twin-screw extrusion and characterized by oscillatory rheometry. It was shown that their behavior could be described by Carreau–Yasuda equations, possibly with a yield stress, in which all parameters depended on the weight average molecular weight. By using these experimental data, it is show in the present study that a time/molecular weight superposition allowed for a drastic reduction in the number of parameters to be considered in order to precisely describe the viscous behavior of these materials. This concept was then validated by applying it to various experimental data from the bibliography.

## 1. Introduction

At the end of the polymerization reactor, polypropylene (PP) resins generally have a high molecular weight and a wide molecular weight distribution, which gives them high viscosity and elasticity values. To make them easier to be processed in extrusion or injection molding, they are chemically modified with peroxides, which break the polymer chains by scission and lead to so-called controlled-rheology polypropylenes (CR-PPs) [1,2,3,4,5,6,7,8]. In addition to the characterization of the kinetic reactions and the development of kinetic models [9,10], the rheological behavior of these CR-PPs has been studied by many authors. Tzoganakis et al. [11] characterized various CR-PPs of different molecular weight distributions. The viscosity curves obtained by capillary rheometry were fitted by power laws or polynomial expressions, the parameters of which were expressed as function of the weight average molecular weight. Later on, the viscoelastic properties of the same samples were quantified using an integral constitutive equation of the K-BKZ type [12]. Tzoganakis [13] also proposed a comparison of the viscoelastic behavior between linear and branched CR-PPs, while Nie and Tzoganakis [8] compared CR-PPs prepared from metallocene and Ziegler–Natta resins. Carrot et al. [14] used the molecular weight distribution (MWD) data of a series of CR-PPs to predict their linear viscoelastic behavior. The inverse problem (i.e., the prediction of the MWD from rheological data in small amplitude oscillatory shear) was treated by Azizi et al. [15]. In a paper published in 2001, Berzin et al. [16] characterized the rheological behavior of CR-PPs, both homo- and copolymers, produced by reactive extrusion. They showed that the viscosity of these materials can be described by a Carreau–Yasuda equation, possibly with a yield stress, of which all the parameters (i.e., Newtonian viscosity, characteristic time, power law index, Yasuda parameter, and yield stress) were, more or less, simple functions of the weight average molecular weight, *M_w_*. In the present paper, the objective was to resume these experimental data and to show how a time/molecular weight superposition, based on the same principle as the classical time/temperature superposition, allows to obtain mastercurves and to propose a general viscosity law for a series of CR-PPs with a reduced number of parameters.

## 2. Materials and Methods

**Figure 1:**
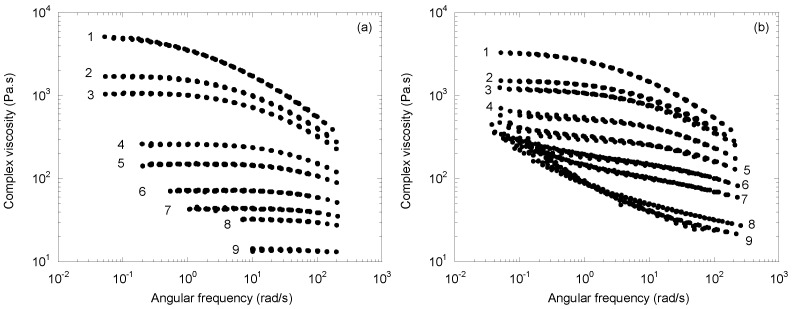
Complex viscosity as a function of angular frequency at 215 °C for (**a**) the homopolymer and (**b**) the copolymer samples. The numbers refer to Table 1 and Table 2.

The results of Berzin et al. [16] concern a series of CR-PPs obtained from a homopolymer and a copolymer containing approximately 7 wt% polyethylene (PE). These samples were prepared by reactive extrusion with different amounts of peroxide (DHBP, Trigonox 101, Akzo Nobel, Amsterdam, The Netherlands) and characterized in terms of molecular weight distribution by steric exclusion chromatography (SEC) [17]. Rheological measurements were performed in the dynamic mode in the linear domain on a rotational rheometer (RMS 800, Rheometrics, Piscateway, NJ, USA) at five temperatures between 185 and 245 °C. Figure 1 shows the complex viscosity mastercurves of the different samples, obtained by time/temperature superposition. The corresponding average molecular weights are indicated in Table 1 and Table 2.

For the homopolymer (Figure 1a), a gradual transition from a Carreau–Yasuda-type behavior to a Newtonian one was observed with a viscosity decrease of almost three decades. This kind of behavior has been reported by all authors who have characterized CR-PPs, either in the dynamic or in the continuous mode [4,11,12,15,18]. For the copolymer (Figure 1b), things were very different: if the viscosity decreased at high frequency when increasing the peroxide content, it increased, in contrast, at low frequency, leading to a crossing of the curves. The complexity of peroxide degradation in ethylene–propylene copolymers has been emphasized by Gahleitner [19]. Whereas when PP chains are broken by β-scissions, a PE-rich phase (i.e., a combination of more or less crystalline ethylene–propylene copolymers) can branch and even crosslink [20,21]. These structural changes induce additional long relaxation times and the onset of an apparent yield stress on the most degraded products [16]. The SEC curves do not exhibit a double distribution, but at 0.5% peroxide, the percentage of chains with an average molecular weight greater than 300 kg/mol is 3.6% for the copolymer versus 0.7% for the homopolymer. Soxhlet extraction in xylene for 24 h does not evidence any gel fraction [22]. The presence of numerous long branches on these initially linear materials is also confirmed by the activation energy values, which increase from 37 to 46 kJ/mol for the homopolymer to more than 60 kJ/mol for the most degraded copolymer [23,24].

Each curve in Figure 1a can be described by a Carreau–Yasuda equation:

(1) $$\left|\eta *\right|=\eta_{0}{\left[1+{\left(\lambda \omega \right)}^{a}\right]}^{\frac{n-1}{a}}.$$

where *η*_0_ is the Newtonian viscosity; *λ* the characteristic time; *n* the power law index; *a* the Yasuda parameter. For those in Figure 1b, an apparent yield stress, *σ*_0_, must be added to reflect the increase in the complex viscosity at low frequency:

(2) $$\left|\eta *\right|=\frac{\sigma_{0}}{\omega }+\eta_{0}{\left[1+{\left(\lambda \omega \right)}^{a}\right]}^{\frac{n-1}{a}}.$$

In the original paper by Berzin et al. [16], the parameters of these laws were fitted for each curve, and the values of *η*_0_, *λ*, *n*, *a*, and *σ*_0_ were expressed as a function of the weight average molecular weight, *M_w_*, by polynomial functions or power laws. This led to complex and tricky forms of the laws, with many parameters. Typically, at least 10 parameters were necessary for one viscosity function. In what follows, we propose a new way of treating these data in order to obtain equally precise but much simpler laws.

## 3. Results

### 3.1. Principle of Time/Molecular Weight Superposition

The basic idea is to consider that a decrease in molecular weight linked to a decrease in the length of macromolecular chains gives them more mobility as would an increase in temperature. In 1966, Vinogradov and Malkin [25] proposed to plot *η*/*η*_0_ as function of $\eta_{0}\dot{\gamma }$ to obtain a “universal” behavior, with *η*_0_ being the Newtonian viscosity. This concept was then applied by Minoshima et al. [26] to polypropylenes of different molecular weight distributions. They showed that the concept worked correctly for materials of a similar distribution width but that polymolecularity had a significant effect. In the field of CR-PPs, to our knowledge, only Barakos et al. [12] have tried this kind of approach, but the results were not totally convincing: the superposition was correct at high shear rate values but failed at low shear rates. In the following, this basic idea is applied to our experimental results in order to obtain mastercurves by using a shift factor, *a_M_*, to superimpose the curves of the different samples.

#### 3.1.1. The Case of a Homopolymer

**Figure 2:**
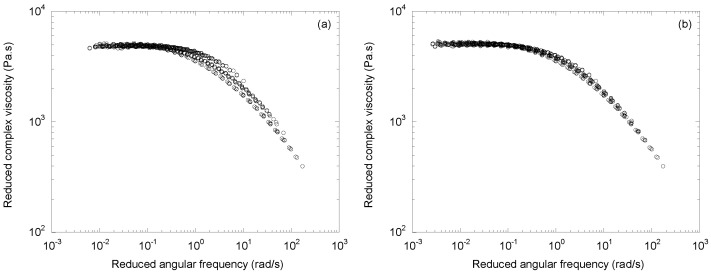
Mastercurves of the complex viscosity at 215 °C for the homopolymer obtained with (**a**) a single shift factor, *a_M_*, and (**b**) two shift factors, *a_M_* and *b_M_*.

Figure 2a shows the result obtained by plotting the reduced complex viscosity |η*|/*a_M_* as a function of the reduced frequency *ω a_M_*, taking the initial polymer as a reference. It can be seen that the superposition is correct at low frequency but with a larger dispersion at the onset of the power law transition. This result, similar to that of Barakos et al. [12], is therefore not totally acceptable.

In fact, by taking the same shift factor for the viscosity and the frequency, it was assumed that the Newtonian viscosity and the characteristic time had the same dependence on the average molecular weight, which is the case, in theory, for linear monodisperse polymers [27]. However, Berzin et al. [16] have shown that this has been not verified for these materials with the following dependencies:

(3) $$\eta_{0}=AM_{w}^{3.9}.$$

(4) $$\lambda =BM_{w}^{4.9}.$$

where *A* and *B* are constants. 

In view of Equations (3) and (4), it seems legitimate to perform a superposition with two shift factors: one *a_M_* for the viscosity and the other *b_M_* for the angular frequency. This gives the result shown in Figure 2b, which is now very satisfactory: a single mastercurve is obtained with 873 experimental points, covering five decades of angular frequency. 

**Figure 3:**
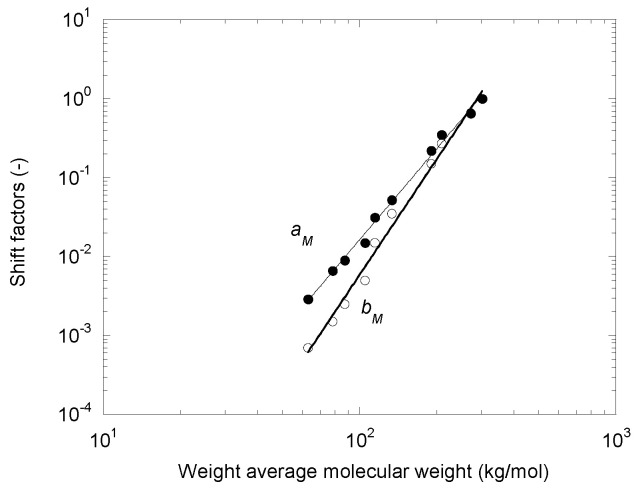
Shift factors *a_M_* and *b_M_* as functions of the weight average molecular weight.

The variations in the shift factors, *a_M_* and *b_M_*, with the molecular weight are shown in Figure 3. 

They both follow a power law evolution:

(5) $$a_{M}=3.5\times 10^{-10}M_{w}^{3.83}.$$

(6) $$b_{M}=1.06\times 10^{-12}M_{w}^{4.87}.$$

where *M_w_* is expressed in kg/mol. For slightly degraded polymers (i.e., those with a low amount of peroxide), the values of *a_M_* and *b_M_* are very close, but the deviation increases as the molecular weight decreases. With *a_M_* and *b_M_* reflecting the dependencies of *η*_0_ and *λ* with the molecular weight, it is normal to find expressions close to those proposed by Berzin et al. [16] and reported in Equations (3) and (4). The exponent equal to 3.83 for the dependence of the Newtonian viscosity was larger than the usual value of 3.4 but lower than that of 4.65 obtained by Carrot et al. [14] on a series of CR-PPs. This was due to the polydispersity of the samples [28], which was lower in our case (i.e., PDI from 6.4 to 2.5) than in that of Carrot et al. [14] (i.e., PDI from 10.6 to 4.6). A difference between the exponents for viscosity and relaxation time can also be observed in the data of many authors. The respective values of 2.2 and 2.9 can be extracted from the results of Barakos et al. [16], and 4.1 and 3.9 from those of Zhang [29].

Finally, the mastercurve in Figure 2b can be fitted by a Carreau–Yasuda equation integrating molecular weight and temperature effects:

(7) $$\left|\eta *\right|=\eta_{0}a_{T}a_{M}{\left[1+{\left(\lambda a_{T}b_{M}\omega \right)}^{a}\right]}^{\frac{n-1}{a}}.$$

where *a_T_* is the temperature shift factor, defined by an Arrhenius law:

(8) $$a_{T}=\exp\left[\frac{E}{R}\left(\frac{1}{T}-\frac{1}{T_{0}}\right)\right].$$

where *E* is the activation energy; *R* is the gas constant; *T*_0_ is a reference temperature; *a_M_* and *b_M_* are defined by Equations (5) and (6). The parameters of Equation (7) are as follows: *η*_0_ = 5010 Pa.s at *T*_0_ = 488 K (215 °C); *λ* = 0.28 s; *a* = 0.7; *n* = 0.40. For the Arrhenius law, one can choose an average value of the activation energy *E* (here, *E* ≈ 45.7 kJ/mol) or consider its slight dependence on the molecular weight [16].

Finally, we end up with a very simple form of law with a limited number of parameters, allowing for the accurate and complete description of the rheological behavior of homopolymer CR-PPs.

#### 3.1.2. The Case of a Copolymer

**Figure 4:**
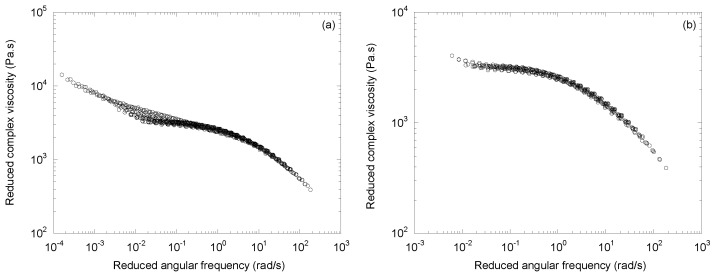
(**a**) Mastercurve of the complex viscosity at 215 °C for the copolymer obtained with two shift factors, *a_M_* and *b_M_*, and (**b**) the mastercurve of the complex viscosity at 215 °C for the slightly degraded copolymer obtained with two shift factors, *a_M_* and *b_M_*.

One could imagine carrying out the same type of treatment on the copolymer to exploit the curves of Figure 1b. The results are presented in Figure 4a. 

As expected, the superposition was not satisfactory. Indeed, as indicated previously, the rise in the viscosity at low frequency was linked to a significant modification of the copolymer’s structure. The effect of peroxide on the structure of ethylene–propylene copolymers was studied by the team of van Reenen [30,31], but an accurate description of the molecular structure of the samples is beyond the scope of this paper. Nevertheless, it is clear that it is illusory to try to obtain a mastercurve in the case of the copolymer. However, it can be seen in Figure 4b that if only the slightly degraded samples that did not show a marked yield stress (samples 1 to 4) are considered, the superposition principle works as well as for the homopolymer. The shift factors also follow the power laws such as those in Equations (5) and (6) but with slightly different exponents, respectively, 3.74 for *a_M_* and 4.66 for *b_M_*.

### 3.2. Validation of the Data in the Literature

Some examples of experimental flow curves for CR-PPs of various molecular weight distributions, either in the dynamic or continuous mode, can be found in the literature. To validate our approach, the previous protocol was applied to them. Even though it is known that treating the results of rheological tests carried out in different laboratories may be difficult, we see that, in the present cases, it worked rather well.

**Figure 5:**
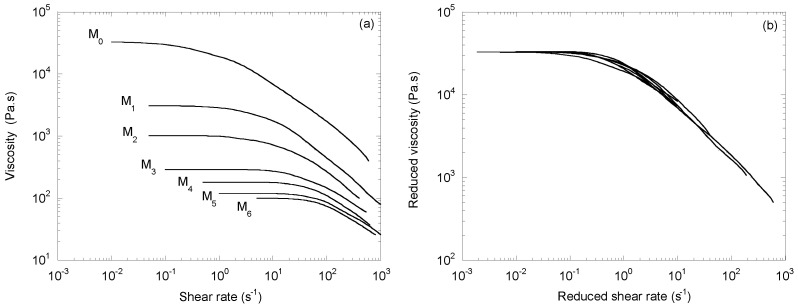
(**a**) Viscosity as a function of the shear rate at 230 °C for a series of CR-PPs (adapted from [4]), and (**b**) the mastercurve obtained with two shift factors, *a_M_* and *b_M_*.

In 1999, Hammerschmid and Gahleitner [4] proposed viscosity curves for six CR-PPs (M_1_ to M_6_) obtained from a homopolymer, M_0_, with peroxide levels ranging from 0.026 to 0.24 wt% (Figure 5a). The shapes of the curves were similar to those in Figure 1a. Figure 5b shows that the time/molecular weight superposition principle with two shift factors also worked correctly. As in the previous examples, *a_M_* and *b_M_* followed power laws with respective exponent values of 3.35 and 2.83.

**Figure 6:**
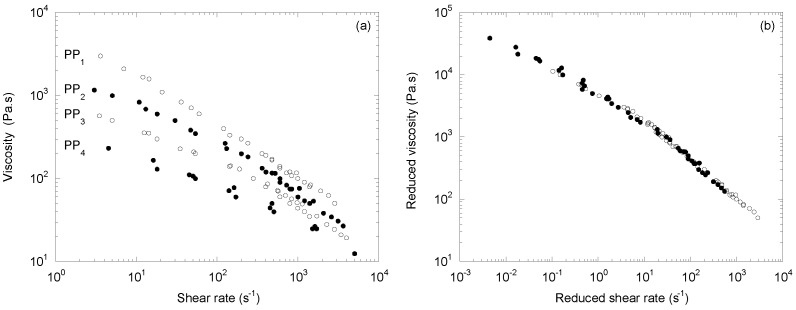
(**a**) Viscosity as a function of the shear rate at 210 °C for a series of CR-PPs (adapted from [12]), and (**b**) the mastercurve obtained with two shift factors, *a_M_* and *b_M_*.

Barakos et al. [12] also published viscosity curves obtained by capillary rheometry for a linear polypropylene PP_1_ and three CR-PPs (PP_2_ to PP_4_) made with peroxide levels from 0.02 to 0.1 wt%. Figure 6a shows the curves of the four products obtained by time/temperature superposition at 210 °C of the data measured at 190, 210, and 230 °C. Compared to the previous examples, the range of shear rates was higher, which explains the absence of a Newtonian plateau. 

Figure 6b shows that the proposed superposition principle still applied perfectly but with higher values for the dependence of the *a_M_* and *b_M_* shift factors on the molecular weight, respectively, at 5.33 and 7.06. This can be explained by the fact that the materials of Barakos et al. [12] had a very high polydispersity index, from 11 for PP_1_ to 4.9 for PP_4_, while it was much lower for Hammerschmid and Gahleitner [4] (i.e., from 5.5 to 2.5) and for Berzin et al. [16] (i.e., from 6.4 to 2.5).

**Figure 7:**
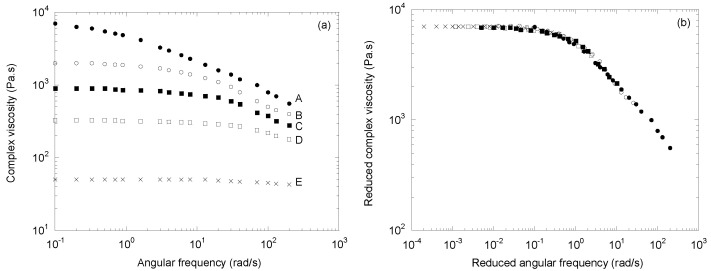
(**a**) The complex viscosity as a function of angular frequency at 230 °C for a series of CR-PPs (adapted from [15]), and (**b**) the mastercurve obtained with two shift factors, *a_M_* and *b_M_*.

In Figure 7a, we plotted the complex viscosity curves obtained by Azizi et al. [15] on a PP homopolymer (A) and four CR-PPs (B to E) obtained with peroxide levels from 0.02 to 0.6 wt%. Figure 7b confirms, once again, the validity of the time/molecular weight superposition concept, with the respective exponents of 4.95 and 6.23 for *a_M_* and *b_M_*. These high values for a polydispersity index close to 2 are perhaps related to the fact that in this paper, the molecular weights were not measured but estimated from rheological data.

**Figure 8:**
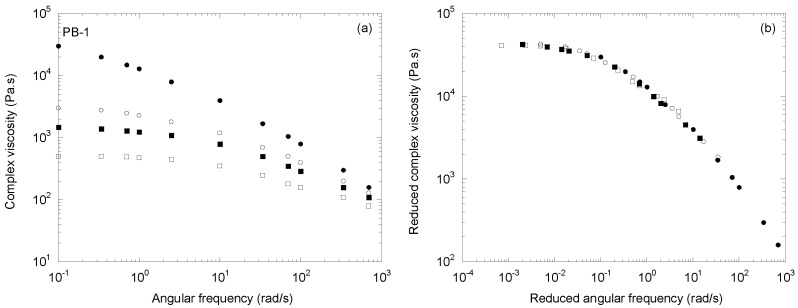
(**a**) The complex viscosity as a function of the angular frequency at 190 °C for a series of CR-poly(1-butene)s (adapted from [29]), and (**b**) the mastercurve obtained with two shift factors, *a_M_* and *b_M_*.

Finally, the concept was applied to another type of linear polymer. Indeed, the peroxide degradation by reactive processing was recently applied by Zhang et al. [32] to poly(1-butene) resins in order to produce tailor-made controlled-rheology poly(1-butene). Figure 8a presents the complex viscosity curves of the initial PB-1 and the three degraded polymers obtained with 0.02 to 0.08 wt% peroxide. The global shapes of these curves were similar to those of usual CR-PPs. Figure 8b shows that the time/molecular weight superposition may also be applied to this new type of polymer with the same success. The indices of the power laws describing the change in *a_M_* and *b_M_* with the molecular weight were 5.4 and 6.1, respectively.

## 4. Conclusions

We showed that the viscosity curves of peroxide-degraded homopolymer polypropylenes of various molecular weight distributions can be gathered into a single mastercurve using two shift factors depending on the weight average molecular weight: one for the Newtonian viscosity and the other for the characteristic time. This principle also applied to the case of copolymers as long as they were not too degraded, i.e., before the appearance of an apparent yield stress. In fact, this principle held as long as the PP had a single-phase structure and the shape of the molecular weight distribution was similar. The two shift factors obeyed power laws as a function of the molecular weight with different exponent values. This principle of superposition, established on our own previous data, was then validated by applying it to the results from the bibliography, both for polypropylene and poly(1-butene) resins. This allowed for the definition of viscous laws, where the viscosity is a function of the shear rate, the temperature, and the weight average molecular weight with a reduced number of parameters.

## Figures and Tables

**Table 1:** The average molecular weights and polydispersity index (PDI) of the homopolymer samples and the corresponding peroxide amount.

Sample	1	2	3	4	5	6	7	8	9
Peroxide (wt%)	0	0.01	0.02	0.06	0.10	0.15	0.25	0.35	0.50
*M_w_* (kg/mol)	301.6	209.3	190.5	135.6	114.8	104.9	87.5	78.8	63.0
*M_n_* (kg/mol)	47.1	45.9	40.6	36.2	34.7	33.6	29.1	27.2	25.2
*M_z_* (kg/mol)	1125.0	482.3	425.8	278.1	224.8	202.6	175.5	148.3	112.6
PDI (-)	6.4	4.6	4.7	3.7	3.3	3.1	3.0	2.9	2.5

**Table 2:** The average molecular weights and polydispersity index (PDI) of the copolymer samples and the corresponding peroxide amount.

Sample	1	2	3	4	5	6	7	8	9
Peroxide (wt%)	0	0.01	0.02	0.06	0.10	0.15	0.25	0.35	0.50
*M_w_* (kg/mol)	256.1	210.4	181.4	168.9	150.1	116.9	104.7	89.3	74.4
*M_n_* (kg/mol)	47.1	44.8	41.8	36.7	36.1	30.0	29.8	24.0	21.8
*M_z_* (kg/mol)	850.5	559.7	456.0	612.3	488.9	285.4	305.0	218.9	231.7
PDI (-)	5.4	4.7	4.3	4.6	4.2	3.9	3.5	3.7	3.4

## Data Availability

Not applicable.
